# Is the Institutional Environment a Challenge for the Well-Being of Female Managers in Europe? The Mediating Effect of Work–Life Balance and Role Clarity Practices in the Workplace

**DOI:** 10.3390/ijerph15091813

**Published:** 2018-08-22

**Authors:** Deybbi Cuéllar-Molina, Antonia M. García-Cabrera, Ana M. Lucia-Casademunt

**Affiliations:** 1 Department of Business Administration, Universidad de Las Palmas de Gran Canaria, Juan de Quesada, 30, 35001 Las Palmas de Gran Canaria, Spain; deybbi.cuellar@ulpgc.es (D.C.-M.); antonia.garcia@ulpgc.es (A.M.G.-C.); 2 Department of Business Management, Universidad Loyola Andalucía, 14004 Cordoba, Spain

**Keywords:** employee well-being, human resource practices, institutional theory, female managers, European countries

## Abstract

The advancement of women to top management positions positively affects firm competitiveness. However, this advancement may also negatively affect individuals as women find themselves forced to overwork to match their male counterparts in organisations, which can cause a decrease in their professional well-being. Although the literature highlights that human resource practices (HRPs) have a positive impact on well-being, it also warns that national institutions may condition the adoption of HRPs by organisations. If that is true, institutions may become either a challenge to—or trigger for—female managers’ well-being. Accordingly, this study analyses the effects of institutions and the mediating effects of HRPs on the influence that is exerted by institutions on well-being. The empirical analysis, which was carried out on a sample of 575 female managers located in 27 European countries, confirms the direct and indirect effects (through HRPs for work–life balance and role clarity) of institutions on female managers’ well-being at work.

## 1. Introduction

The participation of women in the labour market no longer represents a separate or peripheral part of the labour force. However, there is only a minority of women who reach top positions [1], mainly because they face many obstacles that are related to gendered career paths [2]. The access for women to managerial positions has a direct impact on their personal and professional conditions; e.g., the likely lack of work–life balance and role clarity at such positions substantially modifies their immediate work environment and thus conditions female managers’ well-being. First, women may find themselves forced to extend the working day to unbearable limits, even giving up their leisure time. Overwork in the long term is unsustainable, with serious consequences for female manager’s quality of life and well-being [3]. Second, a lack of role clarity can be regarded as another obstacle to female managers’ well-being, because managers’ positions tend to be broadly designed, and hence managers are expected to be responsible for all of the functions and tasks in their area of influence. Although the study of role clarity has mainly been focused on employees’ posts, for female managers it can be highly relevant because a lack of role clarity represents a situational stressor, which can result in the experience of reduced well-being as they struggle to keep up in their managerial positions.

In addition, the previous literature shows evidence of significant differences in how working conditions are implemented into HRPs in organisations in different countries [4], because the national institutional conditions might favour the implementation of a specific human resource practice (HRP) [5]. Along this line, from the new institutional theory perspective, institutions include regulative, normative, and cognitive elements and activities that provide social behaviour, balance, and purpose [6], and thus they influence the decisions that are made by managers and lead organisations to embrace similar practices [7]. Specifically, regulative institutions refer to laws that exist in a given country; normative institutions are closely related to the cultural context, and cognitive institutions emphasise cognition and actors’ common perceptions of behaviours that are conventional or assumed [6]. Since the national institutional framework is different in each country, it is said that organisations must use organisational HRPs to facilitate them to adapt to their context [8]. When this is the case, and we accept the premise that HRP conditions employee well-being [9], it can be expected that national institutions affect well-being, either directly or indirectly, through their impacts on the implementation of HRPs in organisations. Therefore, it is suitable to study and deeply understand the institutional factors that determine how organisations use and develop the HRPs that affect female managers’ well-being. Based on the above, the current objective is to analyse the effect of institutions on female managers’ well-being and particularly the mediating impact of work–life balance (WLB) and role clarity that HRPs provide in that relationship. Along this line, a European cross-national sample is highly appropriate for this study, since diverse institutional factors cause differences between European countries in terms of HRPs [10].

This study offers three main possible contributions. First, we analyse the potential effect of institutions on the female managers’ well-being. Indeed, as far as we know, no previous study has analysed the mediating role of HRPs, such as work–life balance or role clarity, in the effects that are exerted by institutions on female managers’ well-being. Certainly, there are studies on gender differences in well-being [11], and there are also many studies that prove the importance of HRPs for employees in general. In addition, there are diverse studies that analyse the importance of institutions in the design of HRPs in organisations. Thus, the current research could progress our knowledge of the antecedents of well-being, since we assess the specific institutional conditions that define such well-being for this particular group of employees. We specifically analyse the configurations of regulative, normative, and cognitive institutions. Second, we study the impact of institutions on the design and implementation of HRPs in organisations. This analysis allows us to establish whether institutions at the national (country) level determine WLB and the job design domain (role clarity). Third, and more specifically, this study analyses the mediating role of HRPs regarding the effects that institutions exert on female managers’ well-being. This line of analysis may offer new evidence of relationships between institutions and female managers’ working conditions in Europe.

## 2. Theoretical Background and Research Hypotheses

### 2.1. Well-Being at Work

Well-being can be defined as the group of employees’ attitudes and experiences lived within the organisation [12]. From a hedonic approach, it can be understood in terms of the concept of subjective well-being (SWB), that is, individuals’ cognitive and affective evaluations of their lives [5], and in particular, their satisfaction with important domains (e.g., job satisfaction). With reference to this well-being, the present work assumes the social production function (SPF) theory [13]. This theory is built on the premise that people produce their own sense of well-being based on the set of demands and resources that they face at work, and particularly how they employ adaptive strategies to optimise their well-being.

Related to this, a second theoretical approach is useful to ground the current study: the job demand–resource (JD–R) model [14]. This model is suitable for understanding job stress in relation to work environment, and it can be used to improve well-being. It can also be applied to a wide range of occupations [15], among them women’s managerial posts. For this model, job demands refer to the aspects that negatively affect individuals in a variety of life contexts [16]; in terms of work-related factors, they include issues such as work–life conflict and role ambiguity. Conversely, job resources facilitate employees’ family relationships, enhance their quality of life, and improve their emotional states [17].

The basic tenet of the JD–R model is that both job resources and job demands make employees’ day-to-day work either easier or more difficult. Thus, the JD–R model assumes that well-being is essentially conditional on a balance between job resources and job demands. From this framework, when the number of demands matches or is close to the number of resources, employees experience positive emotional states that result in a high level of well-being [18]. Thus, based on this model, it can be stated that organisations may act on female managers’ SWB by using HRPs to improve their job resources. Among them, the present paper focusses on work–life balance (WLB) and job design in terms of role clarity, which are considered to be the enabling psychosocial resources that can potentially buffer the impact of job demands on the job strain [19] that characterises a female manager’s job.

Work–life balance (WLB) refers to the balance between work, family commitments, and personal life [20]. WLB is a form of interrole balance in which both demands are compatible. The ability to balance such domains contributes to well-being at the individual level, the household level, and the social level [21]. In addition, and in line with role theory [22], Kahn et al. [23] defined *role clarity* as “the degree to which individuals feel they have clear guidance about expected roles and behaviours associated with their job”. This domain of job design is defined as the degree to which an individual understands the expected behaviours that are associated with their job and demanded in order for them to fulfil the requirements of their role. These HR practices and their impact on female managers’ subjective well-being are discussed below.

### 2.2. HRPs Regarding Work–Life Balance and Role Clarity and Their Effect on Female Managers’ Subjective Well-Being

Given that most employees dedicate the majority of their time to their work and families, and these are the two vital spheres in most adults’ lives, the research into the links between both has been greatly intensified. Obstacles to family life derived from work tend to be greater than in the opposite direction [24]. Bruck et al. [25] claim that with the longer periods of time that are spent in the workplace, it is likely that conflicts will arise between the work and family domains. Moreover, there is greater pressure from employers who require higher employee involvement and employees who prioritise their professional life over their personal life [21]. Thus, organisations should implement work–life balance HRPs that allow employees to attend to both domains and minimise the conflicts between them [26].

The rival strains between work and family roles often result in conflict for both genders [27]. The particular case of top positions could be further complicated by the associated trend of working long hours and the high levels of responsibility and demands at work [28]. Although family responsibilities are shared more between men and women these days, women are often still mainly responsible. This could be particularly demanding for female managers, as they handle long working hours, even during holidays or on weekends. WLB represents one of the major obstacles (i.e., job demands) that impede the career advancement of female managers, even for women who do not have children. According to Drew and Murtagh [29], one of the greatest challenges related to work–life balance HRPs for female managers is to make the “long hours” culture compatible with having a social and family life. Working long hours usually demonstrates commitment to an organisation, and can be accepted as a basic prerequisite for promotion. If family responsibilities do not interfere, both men and women can compete on an even plane. However, in this regard, senior management culture has been designed and developed for men, and, consequently, it can have a negative impact on female managers. For example, in low egalitarian countries, ratings of female managers’ WLB were significantly lower than those of their male counterparts. WLB is especially complicated for women whose husbands have followed the “breadwinner” model by delegating family and caring activities to them [29]. Moreover, aspects of women’s nature represent an important variable that may further complicate such circumstances. That is, “women’s life-cycle patterns of work and childbearing are diametrically opposed to the senior management career life-cycle. The career stage, when the workload and commitment necessary to succeed are most intensive, coincides with peak child-rearing years” [29].

Previous studies have found strong evidence that work–life issues are critical to ensuring adequate levels of satisfaction and well-being [30], and they can be reached through the use of HRPs for WLB that guarantee such balance as a job resource. A good work–life balance facilitates job satisfaction and has direct effects on outcomes such as well-being [31,32]. Similarly, work–life imbalance could generate dissatisfaction for employees [21]. In fact, Galabova and McKie [33] consider WLB the personal dimension of the construct of well-being. In particular, if employees sense that they have enough time both for their work and social life, it positively impacts on their well-being, as personal wishes and requests are satisfied within that time. Conversely, an unbalance between work and social life negatively affects well-being, since it ends in feelings of frustration [33]. Consequently, it can be expected that:

**Hypotheses** **1** **(H1).** *The greater the level of use of HRPs for work–life balance by organisations, the higher the degree of female managers’ well-being.*

The knowledge of what is expected of an individual in the workplace is essential to the achievement of performance goals. According to Alok et al. [34], receiving clear information about responsibilities and roles at work is essential, because it will in turn help build confidence among colleagues. In many cases, role clarity or ambiguity can be related to the presence or absence of adequate role-relevant information, which is due either to the restriction of such information or to variations in the quality of such information. The restriction of information could represent a relevant barrier that prevents female well-being in senior top management. Role clarity represents a job resource, particularly because female executives still often occupy token status [35] in many organisations, and the positions that are supposedly suited for men are thus more hostile to women’s self-confidence. This domain of job design has a significant positive impact on work outcomes such as well-being [36]. In addition, and more and more frequently, managers are concerned with role involvement, and they seek to design more enriched jobs that include higher role clarity. Furthermore, when jobs are properly designed, they often provide individuals with clear information about their responsibilities within the organisation. This will clearly increase their well-being. Therefore, it is posited that:

**Hypotheses** **2** **(H2).** *The greater the level of use of HRPs for role clarity by organisations, the higher the degree of female managers’ well-being.*

As we have discussed, the literature on HRPs and well-being suggests the existence of positive correlations between them, which we extend to the particular case of WLB and role clarity for female managers. Additionally, as a country’s national institutional environment may condition the design and implementation of the policies and practices that are applied by firms, which are established in each country [8], it is worth studying the effects of institutions on female managers’ well-being through the mediating role of HRPs.

### 2.3. Institutions as the Antecedents of Work–Life Balance, Role Clarity, and Female Managers’ Well-Being

The neo-institutional literature focusses on how an organisational practice may end up as a specific practice within vast “institutional fields” through its imposition, by its social legitimisation or by its imitation [37]. Consequently, organisations that operate in analogous contexts use similar practices and become isomorphic [7].

Scott [6] distinguishes between regulative, normative, and cognitive institutions. The first one refers to rules that exist in a national environment; the second one is more related to the cultural dimension (values that are socially common); finally, the cognitive dimension highlights actors’ shared perceptions of what is standard or taken for granted [6]. The effect of institutions on organisations is due to three distinctive institutional pressures. First, regulative institutions legally force firms to assume specific practices: coercive pressures [6]. Second, as normative institutions determine both socially desirable goals and the appropriate forms through which to reach them (e.g., overwork), managers’ decisions are guided by both self-interest and social consciousness: normative pressures [6]. Third, as organisations confront shared challenges in the national context where they are established and diminish the uncertainty that is caused by such challenges [6], they implement normal solutions: mimetic pressures [38].

However, Caldas and Wood [37] state that institutional factors have been commonly discussed in the literature with regard to management fads and fashions, and the relevant previous studies have provided support for the existence of institutional effects on human resource management [5]. In the human resource field, and from this approach to institutionalism, organisations must assume that external institutions are a given, and hence they must adapt their HRPs to the conditions of such environments [39]. For example, national normative institutions may limit the intensity to which employees deal with the clash between work and family roles [40], and hence exert different pressures on organisations to use work–life balance HRPs. When normative institutions’ approach to uncertainty avoidance is low, uncertain situations do not provide anxiety for employees, and their requirement to avoid risks drops [41]. In this case, employees could put into practice their personal discretionary behaviour to manage work–life balance, thus increasing their perception of well-being [42]. Thus, authors highlight the need for new research to find out whether the knowledge of work–family balance can be extrapolated to different institutional frameworks or if it is characteristic to particular institutions’ frameworks [40]. Similarly, job design, and in particular role clarity, are nested within the organisational context, which is further nested within the external environment, including institutional factors [43]. Thus, the adoption and promotion of job design practices such as role clarity among and between different institutional fields can also be affected by institutional rules, norms, and structures [44,45], such as for example, flexibility and openness, as normative institutions in a country may lead organisations to reduce the use of HRPs to formalise job content through a strict description of tasks.

Although the literature has mainly focused on the analysis of regulative, normative, and cognitive institutions as individual dimensions [39], new institutionalism suggests that the three above-noted institutional dimensions (i.e., regulative, normative, and cognitive) reciprocally reinforce each other [46], as they are interconnected [47] and take the form of the configuration of institutions. Therefore, we state:

**Hypotheses** **3** **(H3).** *The more the configuration of coherent regulative, normative, and cognitive institutions in a given country is favourable to the implementation of work–life balance and role clarity HRPs, the more the use of such practices by organisations that are established in that country.*

Considering the relationships that are discussed above, the configuration of institutions may have an indirect effect on female managers’ well-being by determining the use and implementation of HRPs regarding WLB and role clarity. These HRPs may be the main channel through which the institutional factors influence female managers’ well-being at work. Thus, it can be stated as a mediating hypothesis that:

**Hypotheses** **4** **(H4).** *The implementation of work–life balance and role clarity HRPs are mediator variables in the relation between the configuration of institutions in a specific country and the degree of female managers’ well-being in that country.*

**Figure 1:**
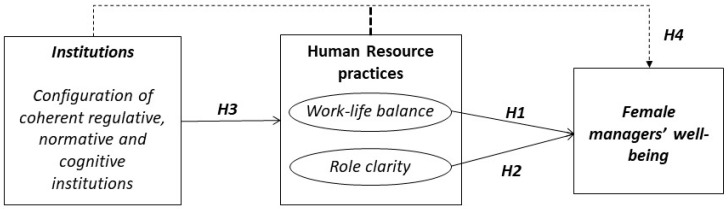
The effects of institutions and the mediating effects of human resource practices (HRPs) on the influence that is exerted by institutions on female managers’ well-being: proposed model.

Figure 1 shows the proposed research model.

## 3. Research Methodology

### 3.1. Data Sources and Study Context

We examine the institutional impact on HRPs and manager well-being, combining individual-level data with country-level data at the international level. Specifically, we focus on the relationship between aspects of the job conditions at the European level that are linked to human resource management (HRM) and the set of national policies and indicators that characterise the environment in which organisations coexist.

The individual-level data was obtained from the fifth European Working Conditions Survey (EWCS-2015) throughout the 27 European Union (EU) member states. The country-level data was obtained from the World Competitiveness Yearbook (WCY-2015). The WCY [48] offers data from 58 countries, although only 27 of them are included in the EWCS [49].

### 3.2. Sample and Research Procedures

We selected a sub-sample of 575 female managers who work for others in 27 European countries (i.e., we disregarded self-employment). Later, the information regarding the country’s regulative, normative, and cognitive institutions from the WCY [49] was aggregated to each selected participant in the EWCS [49]. According to the EWCS [49], the distribution of employment in the EU28 by occupation in 2015 is as follows: Agricultural workers; Managers; Plant and machine operators; Elementary occupations; Clerks; Craft workers; Technicians; Service and sales workers; and Professionals. From them, we chose only the category of Managers, and stated a filter to select females. The female managers had reached the ‘first stage of tertiary education’ (64.2%), and their average age was 42.91. On average, they had worked for more than 10 years at their company or institution. Nearly a third, 32.7%, of female managers were concentrated in medium-sized organisations of the private sector (58.7%), while 31.7% of the subjects worked in the public sector. Furthermore, most of the female managers were located in Belgium (13.4%) and the United Kingdom (9.4%), followed by Norway (8.5%).

Significant differences were found between the mean values for both WLB and role clarity in a full sample of managers and in a sample of female managers. Therefore, these results justify the convenience of studying national institutions as determinants of the use of HRPs to increase SWB.

### 3.3. Measures

#### 3.3.1. Dependent Variable

Subjective well-being was measured using three items that are included in the EWCS [49]: (a) How have you have been feeling over the last two weeks—I have felt cheerful and in good spirits; (b) How have you have been feeling over the last two weeks—I have felt calm and relaxed; and (c) How have you have been feeling over the last two weeks—I have felt active and vigorous. These variables were chosen to measure job-related well-being in line with one of the most accepted models of mental health by [50], which focuses on psychological well-being: an individual’s self-described joy, including positive states such as enthusiasm or cheerfulness, as well as for being considered as part of the World Health Organisation (WHO) model to elaborate a well-being index based on the eudemonic approach [51]. Warr’s (1987) [50] model of well-being is especially useful for the present research, as it is based on the premise that well-being is conditioned by environmental psychological features, such as job characteristics. “Given that people spend a significant proportion of their lives at work, changes in the work environment can have a profound influence on their health and well-being” [52]. A factor analysis was carried out (principal component estimation) with varimax rotation. The Kaiser–Meyer–Olkin (KMO) test and Bartlett’s Test of Sphericity (χ^2^) both offer satisfactory results (KMO = 0.704, χ^2^ = 1566.171, *p*-value = 0.000). The variance explained rises to 73.011% (α = 0.812).

#### 3.3.2. Mediating Variable

As it is possible that those who make decisions on the application of HPRs and those who are affected by them can have different perceptions on the practices that are implemented, we gathered information about HRPs from the perspective of those who bear the brunt of HRPs, that is, the female managers in the sample. Even though many researchers have studied HRPs, individual experiences and perceptions about HRPs have received less empirical attention in the HRM literature [53]. Among their main conclusions, it can be highlighted that there is a gulf between the views of those who implement HRPs and those who are affected by them. Based on the above, our study measures the HRPs for work–life balance and job clarity that are adopted by organisations from the female managers’ viewpoint. Work–life balance was measured as follows: “In general, do your working hours fit in with your family or social commitments outside work very well, well, not very well, or not well at all”? Role clarity was measured as follows: “For each of the following statements, please select the response that best describes your work situation. You know what is expected of you at work”.

We confidently use single items, because studies have analysed the validity of single-item measures, and their findings provide qualified support for them [54,55]. According to Wanous and Hudy [56], the use of a single-item scale to capture the constructs that are under study has demonstrated the ability to validly predict outcomes. Moreover, our review of the empirical literature ratifies the use of a single variable in the study of HRPs through employees’ perceptions as independent variables, both to examine each HRP in isolation [57] and integrate them as a bundle [58,59]. Other studies that examine HRPs as dependent variables have also measured them through one-item scales [60].

#### 3.3.3. Independent Variables

The institutions were measured using the indicators from the WCY. We used seven indicators of regulative aspects: political transparency, fiscal policy, judicial system efficiency, the legal framework for competitiveness, finance and banking regulation, restrictions to foreign organisations, and labour regulations. The seven indicators of normative institutions were: political responsiveness to economic challenges, bureaucratic corruption, bureaucratic hindrance, value system support competitiveness, labour productivity, the flexibility of individuals when they are faced with challenges, and a national culture that is open to foreign ideas. Finally, cognitive institutions were approached by the adaptability of companies to market changes, the entrepreneurship of managers, customer emphasis, technological cooperation, employee training, the productivity of companies that are supported by global strategies, and corporate values that take into account employee values. All of these indicators are coherent with the theoretical bases that are provided by institutionalism for the conceptualisation of the regulative, normative, and cognitive dimensions of a country’s institutional environment. The scale that is used is internally consistent (α = 0.746). A factor analysis (principal component estimation with varimax rotation) was run to identify the configurations of the regulative, normative, and cognitive institutions (KMO = 0.706; χ^2^ = 1,133,205.688, *p*-value = 0.000). The variance explained rises to 85.06%, and four factors were obtained, which described different institutional configurations that accounted for 26.84, 24.97, 17.20 and 16.05 percent of the variance, respectively. The scales that were used to measure each of the obtained institutional configurations have internal consistency (α = 0.668, 0.935, 0.882, and 0.824, respectively). The standardised values (mean is zero and standard deviation is one) of the factors that were obtained from the factor analyses were used in the regression analyses to test the hypotheses.

The first factor was termed “*Private institutions looking at the internal organisation*”, as it included several business practices that point towards developing internal resources and reaching peak productivity (e.g., labour productivity, productivity supported by global strategies, corporate values that respect employee values, and employee training as a priority). The second factor was named “*Public institutions supported by authorities’ practices*” and it was oriented towards boosting business competitiveness (e.g., laws to encourage firms’ competitiveness, political transparency, a lack of unnecessary bureaucracy that harms business operations, political reaction to face economic challenges). The third factor was termed “*Public institutions supported by society that promote flexibility and openness*” with the purpose of supporting firms’ competitiveness (e.g., national cultural values open to overseas thinking, peoples’ flexibility to overcome challenges, and acceptance of foreign firms). The last factor was named “*Private institutions looking at external settings*”, as it encompassed several institutional indicators related to firms’ actions in the environment (e.g., the entrepreneurial behaviour of managers, a firm emphasis on the clients and buyers, and firms’ adaptability to market fluctuations).

#### 3.3.4. Control Variables

The current research included two groups of control variables. At the organisational level, we first used organisational flexibility (4: private sector; 3: Non-governmental organization-NGOs; 2: public–private sector; 1: public sector). We assume that private organisations are more flexible in the adoption of HRPs, while public organisations will have a greater legacy of HR systems. The total number of employees allow us to measure the organisation size variable, as is commonly done in various studies [4]. At the individual level, the age variable was included (measured by the age of the interviewee).

Appendix A shows all of the variables and items used in the current study.

## 4. Data Analysis

In order to examine the possibility of bias due to multicollinearity in the coefficient significance tests, a correlation analysis was carried out between the independent variables. Following this, the test of the hypotheses was developed through multiple linear regressions, which allowed us to analyse the main effect of the independent variables and the mediating effect of HRPs. Collinearity diagnostics were also conducted in linear regressions (variance inflation factor (VIF) and condition number) in order to assess the potential for regression coefficient instability.

To analyse the mediator effect, we followed the method that was proposed by Frazier et al. [61]. (1) Confirm the effect of the predictor (the configuration of institutional factors) on the final outcome variables (female managers’ well-being); (2) Confirm the effect of the predictor on the mediator variable (managers’ perception of the use of WLB and role clarity HRPs); (3) Analyse the effect of the predictor variable and mediator variable on the final outcome variables. If the predictor variable loses its significant effect on the outcome variable when the mediator is introduced into the regression, a full mediator effect exists. However, if the predictor retains its effect on the outcome variables despite the mediator variable being in the regression, only a partial mediation exists.

## 5. Results

In Table 1, note the existence of the highest correlation between *Private institutions looking at external settings* and *age* at—0.199 (*p*-value = 0.000). Moreover, our tests for linear regression (Table 2) show a VIF range from 1.051 to 1.117, which is much lower than the recommended cut-off threshold of 10. The highest condition number for all of the regressions is 14.814, which is lower than the recommended cut-off of 20.

Table 2 shows the regressions estimated to analyse the direct and mediating effects that are described in the hypotheses. Regarding the direct impacts, the results from Model 3 (step 3) confirm hypotheses H1 and H2 and verify the relevance that is exerted by WLB and role clarity HRPs on female managers’ well-being. We identify the positive and significant expected effects (β = 0.215 and 0.109, respectively). Specifically, compared to role clarity, WLB has a more significant capacity to increase female manager’s well-being. These results offer additional evidence of female managers’ greater need for practices to balance work and life to increase their well-being.

To test the third hypothesis, we carried out two regressions to estimate the influence of the configuration of institutions on WLB (Model 1 and step 2) and on role clarity (Model 2, step 2). The results confirm that two out of the four configurations of the national institutions have effects either on one or on the other HRPs. Specifically, the institutional configuration of *Public institutions supported by authorities’ practices* positively affects the use of WLB, but it does not influence the use of role clarity, which is independent of this set of institutions. In addition, while the institutional configuration related to *Public institutions supported by society that promote flexibility and openness* positively conditions organisations’ use of role clarity, it does not affect the use of WLB. The institutional configurations that are related to *Private institutions looking at the internal organisation* and *Private institutions looking at external settings* do not affect the organisations’ use of any the studied HRPs. These results support hypothesis H3, because configurations of institutions from national regulative, normative, and cognitive institutions that are favourable to the implementation of a specific HRP condition an organisations’ use of these HRPs.

Finally, Hypothesis 4 postulates that configurations of institutional factors influence the well-being of female managers through the implementation of WLB and role clarity HRPs. Considering the results of the previous models and steps, step 3 in Model 3 involved the final estimation to test the mediating role of the HRPs of WLB and role clarity regarding the impact of the configuration of institutional factors on female managers’ well-being. The institutional configuration of *Public institutions supported by authorities’ practices* is the only one that has an effect on both the dependent variable *Female managers’ well-being* (Model 3, step 2) and the mediating variable *Work–life balance practice* (Model 1, step 2). In addition, the institutional configuration that is related to *Public institutions supported by society that promote flexibility and openness* is the only one that has an effect on both the dependent variable *Female managers’ well-being* (Model 3, step 2) and the mediating variable *Role clarity practice* (Model 2, step 2). Therefore, any mediating effect of each HRP is only possible for these particular combinations. The estimation that was carried out in Model 3 (Step 3) indicates that in the presence of the mediating variables, the institutional configuration that is related to *Public institutions supported by society that promote flexibility and openness* loses its significant, positive effect on the final dependent variable. More specifically, the estimated beta coefficients confirm that the two HRPs have a significant, positive effect on job well-being, while the mentioned configuration of institutions loses its significant impact on the dependent variable. Thus, the direct effect of this configuration of institutions on female managers’ well-being (as is shown in Model 3 and step 2) disappears in the presence of the role clarity practice. However, the other configuration of institutions, i.e., *Public institutions supported by authorities’ practices*, keeps its positive and significant effect on well-being in the presence of the WLB practice. Therefore, as predicted, it retains its effect on the dependent variable, despite the mediator variable being in the regression, and there is only a partial mediation. These results indicate that in the first case, the configuration of institutions does not have a direct effect on the increasing of female managers’ well-being; however, it does have an indirect effect over the implementation of practices of role clarity, while in the second case, the institutional configuration also has a direct and indirect impact on female managers’ well-being. These results provide support for H4.

In addition, our results show that the configuration of institutions can directly affect female managers’ well-being. For example, the institutional configuration that is related to *Private institutions looking at the internal organisation* does not influence organisations’ use of WLB and role clarity practices, but it does have an influence on job well-being (step 3 in Model 3). In addition, as we detail above, the institutional configuration that is related to *Public institutions supported by authorities’ practices* has both direct and indirect effects on female managers’ well-being.

## 6. Discussion 

Our research has analysed the impact of institutions on female managers’ well-being, and particularly, on the mediating effect of WLB and role clarity HRPs in such relationships. The results show how these two HRPs positively affect female managers’ well-being. First, this concludes the importance of supporting female managers by making family and work domains more well-matched. This is particularly relevant for those who find themselves forced to face long hours at work. Thus, our results show that providing female managers the flexibility to balance their responsibilities could be a prerequisite for organisations benefitting from the positive work attitudes and behaviours that emanate from employee well-being. Second, our results indicate the convenience of supporting female managers by enhancing role clarity in work objectives and performance expectations and their responsibilities in their managerial posts. This is important, as it will provide female managers with the opportunity to obtain greater knowledge about exactly what is expected from them. Thus, the current research suggests that organisations should be encouraged to use WLB and role clarity HRPs because they have positive consequences on female managers’ well-being and allow them to remain in managerial positions. Consequently, these HRPs can assist organisations in taking advantage of the benefits that are associated with female representation in managerial posts, such as higher organisational competitiveness and performance [62]. In this regard, the notion that WLB and role clarity HRPs should only be oriented towards employees because managers, due to their position in the organisation, must be willing to overwork and give their life to the company, could be a form of myopic thinking. This narrow and limited conception reduces firms’ opportunities to take advantage of female managers’ skills.

In addition, our results indicate that institutional configurations impact organisations’ use of WLB and role clarity HRPs; hence, they encourage the thesis that the national environment defines organisational behaviour. These findings are in line with recent European projects such as PSYRES (Psychological health and well-being in restructuring: key effects and mechanisms) [63], which recognizes the role of national context on employees’ psychological health and well-being and tries to determine which subgroups of employees are at risk of developing psychological health problems and why. Among the four identified institutional configurations, *Private institutions looking at the internal organisation*, *Public institutions supported by authorities’ practices*, *Public institutions supported by society that promote flexibility and openness*, the current research concludes the importance of *Public institutions supported by authorities’ practices*. This institutional configuration has the strongest impact on the development of female managers’ well-being in models that estimate both direct and mediating effects. Specifically, this is a configuration that is based on a legal framework that boosts the competitiveness of organisations, a lack of bureaucracy that hampers business activity, and political responsiveness to economic challenges. All of these institutions provide efficiency and flexibility to organisations and facilitate the implementation of work–life balance HRPs. However, with the increase of well-being, this institutional configuration must be accompanied by two other configurations. First, it must be accompanied by the institutions that are related to *Private institutions looking at the internal organisation* such as corporate values that take into account employee values and the idea that employee training is a high priority in companies for labour productivity. This institutional configuration offers organisations an institutional environment that is favourable to the investment in employees that generates valuable human resources. For example, these institutions may offer female managers who are motivated by the search for work–life balance and a successful professional career the opportunity to achieve both objectives because their organisations are particularly committed to human resources and aim to generate a positive labour relationship with their employees. All of this increases female managers’ well-being. Second, an institutional configuration regarding *Public institutions supported by society that promote flexibility and openness* is also necessary. This configuration considers institutional traits such as: “national culture is open to foreign ideas”, “there is flexibility for people to face challenges”, or “legal restrictions to foreign organisations do not exist”. This group of institutions motivates organisations to implement role clarity practices as a way to define and clarify what is expected from managers and, as a result, allows female managers to accurately understand their functions. Thus, as an effect of these institutions on the use of role clarity, these women may develop an active role in the organisation of their own working day to successfully develop their known and identified responsibilities in the organisation. This will noticeably increase their job well-being, as was found by our mediating test. Otherwise, female managers will likely feel stress and will overwork in response to the assumption that more must be done in order for them to be considered equal to other managers, and thus their job well-being will decrease.

## 7. Conclusions

Generally speaking, our findings support the thesis that there is not much room for organisational discretion. Although some researchers have found that the effect of external institutions on organisational practices can be vague because organisations can respond differently to institutional environments [64], our results indicate that there are national regularities, as many organisations conform to their environment. Indeed, the evidence here indicates that the HRPs that are used by organisations are significantly conditioned by national institutions.

Furthermore, our results go beyond the previous expectations as they show that some institutions can directly affect female managers’ well-being, i.e., *Public institutions supported by authorities’ practices* and *Private institutions looking at the internal organisation*, irrespective of the use of HRPs by organisations. Given this finding, we can formulate some pertinent questions that should be answered: In Europe, are the institutions a real obstacle to increasing female managers’ well-being? If so, what must policy makers do? For example, countries such as Greece, Hungary, Lithuania, Poland, Portugal, the Slovak Republic, Spain, and Turkey have low levels of the two institutional configurations that directly affect female managers’ well-being. Therefore, the institutional environment appears to be a verifiable challenge for the well-being of female managers in these European countries. Accordingly, new questions emerge that should be answered by further research. Do institutions erode organisations’ competitiveness in some European countries? If so, how can organisations in these countries deal with institutions in order to enhance female managers’ well-being?

### Implications of Practice

The current study has several important practical implications. First, because the institutions affect the organisations’ use of HRPs that influence female managers’ well-being, the public administrations could consider legislation and business practices in each industry sector, among others, and examine how they are enforced in a specific country and/or region. Particularly, although regulative institutions can contribute to the encouragement of organisations’ use of HRPs because laws can enforce firm behaviour, this effect is conditioned by the existence of normative and cognitive institutions that interact with legislation to give rise to institutional configurations. Thus, changes in the regulative institutions may not, in isolation, lead to the expected results, and policy makers may need to complement them with others, hence also promoting normative and cognitive institutions. One example would be stating information about the successful past experiences of organisations that encourage female managers’ well-being in the public domain. Second, as this study confirms the importance of national institutions because managers strongly conform to the institutional environment when they design and implement HRPs in their organisations, we recommend an increase in the use of international mobility programmes and specific international training programmes to allow human resource managers to learn about the alternative bundles of HRPs that are used in foreign countries with higher levels of female managers’ well-being.

Finally, the current research is subject to a number of limitations. First, although the data is related to a great number of countries and employees, it was compiled from a variety of (27) European countries. Thus, our results should not be fully generalised without first determining the relevance of the geographical context. Therefore, we suggest examining these findings in comparison with other geographical contexts (e.g., the Arab world, Asian cultures). The second limitation concerns our ability to make causal inferences from the data. This is limited by the use of a cross-sectional design. For example, our findings cannot show how the same female managers would perceive their well-being if institutional changes occurred in their specific countries. Future research with diverse studies about these variables would benefit from a longitudinal research design. In addition, further research is necessary to estimate the contribution of each level of analysis (i.e., the country effect of regulative, normative, and cognitive configurations separate from the organizational effect of HR practices), as well as the possible interaction between variables of these two levels on female manager’s SWB. It is our belief that conducting multi-level models, or hierarchic linear models that consider these different levels of aggregation would shed light on these issues.

## Figures and Tables

**Table 1:** Correlations, means, and standard deviations.

	1	2	3	4	5	6	7	8	9	10
1. Employee well-being	1									
2. Private institutions looking at the internal organization	−0.093 *									
3. Public institutions supported by authorities’ practices and competitiveness	−0.065	−0.054								
4. Public institutions supported by society that promote flexibility and openness	−0.062	−0.018	−0.073 ^†^							
5. Private institutions looking at external settings	0.073 ^†^	−0.158 ***	0.038	−0.182 ***						
6. Work–life balance	−0.262 ***	0.085 *	0.124 **	0.031	−0.060					
7. Role clarity	−0.127 **	−0.067	−0.049	0.110 **	−0.032	0.084 *				
8. Flexibility (public–private sector)	0.011	0.036	0.052	0.098 *	−0.033	0.038	0.019			
9. Organization size	0.054	0.115 **	0.095 *	0.039	−0.053	0.010	−0.091 *	0.029		
10. Age	0.011	−0.199 ***	0.095 *	0.044	0.062	−0.043	0.027	0.149 **	0.050	1
Mean	0.000	0.182	0.275	0.177	−0.066	0.000	0.000	1.57	4.59	42.9
Standard deviation	0.000	0.969	1.13	0.898	0.840	0.947	0.646	0.850	1.85	10.7

*** *p* < 0.001, ** *p* < 0.01, * *p* < 0.05, † *p* < 0.1.

**Table 2:** Results of models estimated and hypothesis tests: female manager sample. VIF: variance inflation factor.

Variables	Model 1 Work–Life Balance	Model 2 Role Clarity	Model 3 Female Managers’ Well-Being
*Step 1: Controls*			
Organizational flexibility (public–private sector)	−0.024	0.052	0.043
Organization size	0.012	−0.096 *	−0.044
Age	0.087 *	0.161 ***	−0.009
*Step 2: Controls + Main effects*			
Organizational flexibility (public–private sector)	−0.013	0.043	0.052
Organization size	−0.010	−0.091 *	−0.072
Age	0.089 *	0.160 ***	−0.014
Private institutions looking at the internal organization	0.082	−0.059	0.101 *
Public institutions supported by authorities’ practices and competitiveness	0.124 **	−0.048	0.118 **
Public institutions supported by society that promote flexibility and openness	0.014	0.082 *	0.091 *
Private institutions looking at external settings	−0.070	−0.045	−0.051
ΔR^2^	2.6%	1.6%	
ΔF	3.674	2.283	
*Step 3: Controls + Main effects + Mediating effects*			
Organizational flexibility (public–private sector)			0.051
Organization size			−0.053
Age			−0.051
Private institutions looking at the internal organization			0.087 *
Public institutions supported by authorities’ practices and competitiveness			0.093 *
Public institutions supported by society that promote flexibility and openness			0.075
Private institutions looking at external settings			−0.031
Work–life balance			0.215 ***
Role clarity			0.109 **
ΔR^2^			5.7%
ΔF			16.323
*F*	2.883	3.975	6.004
Final adjusted R^2^	2.3%	3.6%	7.9%
Condition number	14.780	14.693	14.814
VIF lower–upper limits	1.116–1.078	1.113–1.081	1.117–1.051

*** *p* < 0.001, ** *p* < 0.01, * *p* < 0.05.

## Appendix A. Items for Measuring Variables in the Current Study

**Subjective Well-Being** **(Dependent Variable)**	**Items from the European Working Conditions Survey (EWCS)**	**Codes**
	How have you have been feeling over the last two weeks—I have felt cheerful and in good spirits?	Q87A
	How have you have been feeling over the last two weeks—I have felt calm and relaxed?	Q87B
	How have you have been feeling over the last two weeks—I have felt active and vigorous?	Q87C
**Human Resources Practices** **(Mediating Variables)**	**Items from the EWCS**	**Codes**
Work–life balance	In general, do your working hours fit in with your family or social commitments outside work very well, well, not very well, or not well at all?	Q44
Role clarity	For each of the following statements, please select the response that best describes your work situation. You know what is expected of you at work.	Q61K
**The Institutions** **(Independent Variables)**	**Indicators from the World Competitiveness Yearbook (WCY)**	
Regulative	Political transparency Fiscal policy Judicial system efficiency The legal framework for competitiveness Finance and banking regulation Restrictions to foreign organisations Labour regulations	
Normative	Political responsiveness to economic challenges Bureaucratic corruption Bureaucratic hindrance Value system support competitiveness Labour productivity The flexibility of individuals when they are faced with challenges National culture that is open to foreign ideas	
Cognitive	The adaptability of companies to market changes The entrepreneurship of managers Customer emphasis Technological cooperation Employee training The productivity of companies that are supported by global strategies Corporate values that take into account employee values	
**Control Variables**	**Items from the EWCS**	**Codes**
Organisational flexibility	Are you working in…? 1—the private sector; 2—the public sector	Q14
Organisational size	How many employees in total work in your company or organisation?	Q16b
Individual age	How old are you?	Q2b
